# Persistent Hyperactivity of Hippocampal Dentate Interneurons After a Silent Period in the Rat Pilocarpine Model of Epilepsy

**DOI:** 10.3389/fncel.2016.00094

**Published:** 2016-04-08

**Authors:** Xiaochen Wang, Xinyu Song, Lin Wu, J. Victor Nadler, Ren-Zhi Zhan

**Affiliations:** ^1^Department of Physiology, Shandong University School of MedicineJinan, China; ^2^Department of Respiratory Medicine, Affiliated Hospital of Binzhou Medical UniversityBinzhou, Shandong, China; ^3^Department of Pharmacology and Cancer Biology, Duke University Medical CenterDurham, NC, USA

**Keywords:** activity-regulated cytoskeleton associated protein, c-fos, GABAergic, immediate early genes, neuronal nitric oxide synthase, neuropeptides, parvalbumin, spontaneous firing

## Abstract

Profile of GABAergic interneuron activity after pilocarpine-induced status epilepticus (SE) was examined in the rat hippocampal dentate gyrus by analyzing immediate early gene expression and recording spontaneous firing at near resting membrane potential (REM). SE for exact 2 h or more than 2 h was induced in the male Sprague-Dawley rats by an intraperitoneal injection of pilocarpine. Expression of immediate early genes (IEGs) was examined at 1 h, 1 week, 2 weeks or more than 10 weeks after SE. For animals to be examined at 1 h after SE, SE lasted for exact 2 h was terminated by an intraperitoneal injection of diazepam. Spontaneous firing at near the REM was recorded in interneurons located along the border between the granule cell layer and the hilus more than 10 weeks after SE. Results showed that both c-fos and activity-regulated cytoskeleton associated protein (Arc) in hilar GABAergic interneurons were up-regulated after SE in a biphasic manner; they were increased at 1 h and more than 2 weeks, but not at 1 week after SE. Ten weeks after SE, nearly 60% of hilar GABAergic cells expressed c-fos. With the exception of calretinin (CR)-positive cells, percentages of hilar neuronal nitric oxide synthase (nNOS)-, neuropeptide Y (NPY)-, parvalbumin (PV)-, and somatostatin (SOM)-positive cells with c-fos expression are significantly higher than those of controls more than 10 weeks after SE. Without the REM to be more depolarizing and changed threshold potential level in SE-induced rats, cell-attached recording revealed that nearly 90% of hilar interneurons fired spontaneously at near the REM while only 22% of the same cell population did so in the controls. In conclusion, pilocarpine-induced SE eventually leads to a state in which surviving dentate GABAergic interneurons become hyperactive with a subtype-dependent manner; this implies that a fragile balance between excitation and inhibition exists in the dentate gyrus and in addition, the activity-dependent up-regulation of IEGs may underlie plastic changes seen in some types of GABAergic cells in the pilocarpine model of epilepsy.

## Introduction

In temporal lobe epilepsy, multiple histopathological changes such as loss of neurons, gliosis, synaptic reorganization, granule cell dispersion, and altered neurogenesis are present in the hippocampal dentate gyrus (Majores et al., 2007; Goldberg and Coulter, 2013). Because of these changes, the dentate gyrus has been studied extensively for its roles in the development of epilepsy and propagation of epileptic waves.

In addition to excitatory granule cells and mossy cells, the dentate gyrus also contains GABAergic interneurons, which function to prevent runaway excitation and to control the temporal and spatial dynamics of granule cell firing (Amaral et al., 2007; Hosp et al., 2014). Like GABAergic interneurons in the cortex, GABAergic interneurons in the dentate gyrus are diverse, varying in morphological appearances, neurochemical identities, electrophysiological properties, and axonal projection domains (Han et al., 1993; Mott et al., 1997; Houser, 2007; Hosp et al., 2014; Wang X. et al., 2014). Epilepsy is associated with numerous alterations in hippocampal dentate GABAergic interneurons. In both animal epilepsy models and humans with spontaneous seizures, certain subtypes of dentate GABAergic interneurons, especially those somatostatin (SOM)- and parvalbumin (PV)-expressing cells are reduced (Sloviter, 1987; de Lanerolle et al., 1989; Schwarzer et al., 1995; Morin et al., 1998; Gorter et al., 2001; Sundstrom et al., 2001; Sun et al., 2007; Kuruba et al., 2011). Accordingly, GABAergic neurotransmission is altered (Hirsch et al., 1999; Cossart et al., 2001; Kobayashi and Buckmaster, 2003; Shao and Dudek, 2005) and plastic changes in surviving GABAergic cells also occur (Zhang et al., 2009; Halabisky et al., 2010; Drexel et al., 2012; Houser, 2014). However, due to the vast diversity of GABAergic interneurons and the difficulty in obtaining electrophysiological recordings in a specific subtype of interneurons without biasing toward to record from a sub-subtype that may resist to status epilepticus (SE), many issues related to functional changes in interneurons remain to be explored. One central issue is whether or not surviving GABAergic interneurons in animals with spontaneous seizures become hyperactive in a subtype-dependent manner. The promise of GABAergic progenitor transplantation and activation of GABAergic interneurons through optogenetics in reducing frequency and severity of seizures as well as in improving abnormal behaviors (Calcagnotto et al., 2010; Hunt et al., 2013; Henderson et al., 2014; Southwell et al., 2014; Tyson and Anderson, 2014) that associate with epilepsy raise a question on if specific type(s) of GABAergic interneurons should be transplanted for achieving optimal outcomes.

Neuronal activity such as sensory stimulation, behavioral tasks, or seizures often induces gene expression changes *in vivo*. Genes expressed that do not require protein synthesis are termed as immediate early genes (IEGs). Expression of IEGs either at mRNA and/or at protein levels in turn can be used to profile nerve cell activity (Okuno, 2011; Yang et al., 2014). At least four IEGs, including c-fos (Simonato et al., 1991; Hosford et al., 1995; Scharfman et al., 2002; Dyrvig et al., 2012, 2014), early growth response protein 1 (Egr1, also known as Zif268; Honkaniemi and Sharp, 1999; Szyndler et al., 2013; Dyrvig et al., 2014), activity-regulated cytoskeleton associated protein (Arc or Arg3.1; Link et al., 1995; Lyford et al., 1995; Akiyama et al., 2008; Dyrvig et al., 2012; Szyndler et al., 2013) and neuronal PAS domain protein 4 (Npas4; Wang D. et al., 2014) have been reported to be up-regulated by seizures induced either by electroconvulsive stimuli or through administration of chemoconvulsants. However, problems exist in using above mentioned IEGs to indicate activity of GABAergic cells after SE. First, evidence exists that activity-dependent genes are differentially regulated in different cell types with the same stimulus (Lyons and West, 2011; Kawashima et al., 2014). Second, IEGs may be differentially up-regulated in response to different stimuli in the same cell type. For example, in cultured hippocampal neurons, Arc and c-fos, but not Npas4, were up-regulated by neurotrophic factors (Ramamoorthi et al., 2011; Spiegel et al., 2014). Third, expression of certain IEGs may differ between excitatory neurons and inhibitory GABAergic cells (Vazdarjanova et al., 2006).

Through assessing expression of Arc and c-fos and recording spontaneous firing at near resting membrane potential (REM) of hippocampal dentate interneurons in a rat pilocarpine model of epilepsy, we revealed that after a short latent period, surviving hilar interneurons after pilocarpine-induced SE become persistently hyperactive in a subtype-dependent manner.

## Materials and Methods

### Animals

All animal experiments were approved by the Animal Ethics Committee of Shandong University School of Medicine and performed according to the guides for the care and use of laboratory animals set by the US National Research Council. Sprague-Dawley rats (160–180 g, 6–7 weeks old) purchased from Beijing HFK Bioscience Company (Beijing, China) were placed in a university animal facility for at least 1 week with a 12 h light/dark cycle and free access to food and water. Animals grew to be around 225 g before being subjected to pilocarpine injection or sham treatment.

### Pilocarpine-Induced SE in Rats

SE lasting for at least for 120 min was induced by intraperitoneally injected pilocarpine hydrochloride (Alfa Aesar, Heysham, UK). Pilocarpine was dissolved in normal saline to make a stock solution of 150 mg/ml. Thirty minutes after animals were pretreated with an intraperitoneal injection of methylscopolamine and terbutaline (2 mg/kg body weight each), 150 mg/kg of pilocarpine was injected. Another dose of pilocarpine (300 mg/kg) was given 20 min after the first dose. Methylscopolamine and terbutaline were the products of Sigma (St. Louis, MO, USA). The intensity of seizures was graded according to Racine stages (Racine, 1972). Following the same pretreatment schedule, the controls were given equivalent volume of saline twice at times identical to the pilocarpine-treated animals. Animals were sacrificed at four different points: 1 h, 1 week, 2 weeks or between 10 and 15 weeks (more than 10 weeks) after SE. For those animals to be sacrificed 1 h after SE, SE lasting for exact 2 h was terminated by an intraperitoneal injection of diazepam (5 mg/kg). The controls received an equivalent volume of saline after the same pretreatment and the equal dose of diazepam as those of pilocarpine-injected rats.

### Transcardiac Perfusion and Tissue Preparation

For immunofluorescence experiments, each animal was deeply anesthetized with an intraperitoneal injection of sodium pentobarbital (80 mg/kg). After complete paralysis, the animal was perfused through the left ventricle with heparinized saline, followed by 4% paraformaldehyde in 0.1 M phosphate-buffered saline (PBS, pH 6.8) over a period of 40 min. The brain was removed from the skull upon completion of transcardial perfusion. Tissue block made by trimming off the cerebellum was post-fixed in the same fixative at 4°C overnight. Thereafter, the tissue block was sequentially immersed in 10% sucrose in 0.1 M PBS for 4 h, 15% sucrose in 0.1 M PBS for 8 h, and finally 20% sucrose in 0.1 M PBS at 4°C overnight. After the tissue block was firmly embedded with a medium that consisted of 30% (w/v) chicken egg albumin (Sigma, St. Louis, MO, USA), 0.5% (w/v) gelatin and 0.9% (v/v) glutaraldehyde in 0.1 M PBS as described previously (Wang X. et al., 2014), it was horizontally cut through the hippocampus into 40 μm-thick-sections with a vibratome (VT1000 S, Leica Biosystems, Wetzlar, Germany) to yield serial hippocampal sections. Hippocampal sections with “C” shaped dentate gyri (mainly representing the ventral part of the hippocampal formation) were orderly collected into 0.1 M PBS and used for detecting the expression of IEGs in GABAergic cells in the above mentioned four time points and the activation of different subtypes of interneurons more than 10 weeks after SE. For each rat, three sections (1 in 8 series sections) were picked for staining with the same antibody or antibody pair.

### Double Immunofluorescence

Double immunofluorescence was used to determine if GABAergic interneurons are activated in a subtype-dependent manner by using c-Fos or Arc immunoreactivity as a cellular activity marker. As described above, “C”-shaped horizontal hippocampal sections were prepared from control or SE rats at different time points. After sections were washed with 1XPBS, they were incubated with a blocking solution for 2.5 h at 4 °C to minimize non-specific stains. The blocking solution consisted of 0.2% Triton X-100, 2.5% BSA and 5% donkey serum in 0.1 M PBS. After blocking, sections were incubated with a primary antibody pair that was diluted with the blocking solution at 4°C overnight. The primary antibody pairs included c-fos/Arc, c-fos/GABA, c-fos/GAD_67_, c-fos/calretinin (CR), c-fos/neuronal nitric oxide synthase (nNOS), c-fos/neuropeptide Y (NPY), c-fos/PV and c-fos/SOM. Information of primary antibodies used is given in Table 1. Thereafter, the sections were washed with 1XPBS for three times (15 min each). The Alexa Fluor 488-conjugated donkey anti-goat IgG and Alexa Flour 568-conjugated donkey anti-rabbit IgG (Invitrogen, Carlsbad, CA, USA) in 1:600 dilutions were used as secondary antibodies. The sections were incubated in the secondary antibody mixture for 150 min and then washed with 1XPBS.

**Table 1:** **Information of primary antibodies**.

Antibody	Clonality/Species	Antigen	Manufacturer	Catalog no.	Lot no.	Dilution
Anti-activity-regulated cytoskeleton-associated protein (Arc)	Polyclonal/Rabbit	Strep-Tag® fusion protein of full-length mouse arc	Synaptic system	156-002		1:1000
Anti-calretinin (CR)	Polyclonal /Rabbit	Recombinant rat calretinin	EMD Millipore	AB5054	NG1780667	1:4000
Anti-c-fos	Polyclonal/Goat	Synthetic peptide corresponding to c-fos aa 280-320	Abcam	ab87655		1:2000
Anti-GABA	Polyclonal/Rabbit	GABA-BSA	Sigma-Aldrich	A2052	112M4768	1:1500
Anti-GAD67	Monoclonal (IgG2a) /Mouse	Recombinant GAD67	EMD Millipore	MAB5406	2491208	1:2500
Anti-neuronal nitric oxide	Polyclonal/Rabbit	Synthetic peptide corresponding to nNOS of rat brain (1409-1429) conjugated to KLH	Sigma-Aldrich	N7280	060M4779	1:4000
synthase (nNOS)
Anti-neuropeptide Y (NPY)	Polyclonal/Rabbit	Synthetic peptide	Peninsula Lab	T-4070	A09643	1:4000
Anti-parvalbumin (PV)	Polyclonal/Rabbit	Rat muscle parvalbumin	Swant	PV25	1637	1:6000
Anti-somatostatin (SOM)	Polyclonal/Rabbit	Synthetic somatostatin-14	Peninsula Lab	T-4103	A09211	1:4000

### Confocal Image Acquisition and Analyses

All stained sections were scanned with a Carl Zeiss Laser Scanning Microscope (LSM 780, Jena, Germany). After the dentate gyrus of each section was scanned under a 10× objective with a zoom 0.8, areas were randomly chosen to undergo scanning under a 20× objective (zoom 1). Quantitative counting was done in images that were taken with the 20× objective; all images were taken under the same scanning parameter sets (laser intensity, gain, size and resolution of each frame) for individual immunoreactions. Positive cells were counted under Photoshop (Adobe Photoshop) by darkening hilar background stains. The activation of GABAergic interneurons was determined by the number of GABAergic cells positive for c-fos/the total number of GABA^+^ cells × 100% whereas activations of different subtypes were calculated by the number of marker-expressing cells positive for c-fos/the total number of marker-expressing cells × 100%. Quantitative numbers were averaged in each section first and different sections thereafter in each animal.

### Electrophysiology

Animals were decapitated under deep ether anesthesia at least 10 weeks after SE onset or saline injection. The brain was removed to ice-cold high-Mg^2+^ artificial cerebrospinal fluid (ACSF) that consisted of (in mM) 112 NaCl, 25 NaHCO_3_, 3.1 KCl, 1.8 CaCl_2_, 11.2 MgSO_4_, 0.4 KH_2_PO_4_, 20 D-glucose and equilibrated with 95%O_2_/5% CO_2_. Horizontal hippocampal slices (410 μm in thickness) were cut under a Leica VT1200 S vibrating microtome (Wetzlar, Germany) by using a procedure that was described in a previous publication (Zhan and Nadler, 2009). After cutting, the slices were incubated at 34.5°C for 30 min to promote metabolic recovery. A slice transferred into a submersion-type chamber was immobilized by a platinum ring and continuously perfused with 95% O_2_/5% CO_2_-saturated artificial cerebrospinal fluid (ACSF) that consisted of (in mM) 122 NaCl, 3.1 KCl, 1.8 CaCl_2_, 1.2 MgSO_4_, 0.4 KH_2_HPO_4_, 25 NaHCO_3_ and 20 D-glucose (pH 7.35). Interneuron-like cells along the border of the hilus and granule cell layer were visualized with an Olympus BX51WI system microscope equipped with an infrared optical system, a CCD camera (Qimaging, Surrey, BC, Canada), and a 60× water-immersion objective. Tips were filled with a potassium gluconate-based internal solution that consisted of [in mM: 125 potassium gluconate, 7 KCl, 0.1 EGTA, 2 Tris ATP, 0.3 Tris GTP, 5 creatine phosphate, 20 U/ml creatine phosphokinase, 0.8% (wt/vol) biocytin, pH 7.25–7.30, and 294–297 mOsm]. After a tight seal (≥2 GΩ) was made, spontaneous currents were recorded for 5 min in cell-attached mode at a holding potential of −60 mV. Thereafter, the membrane was ruptured to make whole-cell recording. The REM was determined immediately after break-in. Membrane time constant, input resistance, and membrane capacitance were calculated from the current response to a 10 mV hyperpolarizing voltage from the resting *V*_m_ applied for 200 ms. Evoked firings in response to stepped current injection were recorded in a current-clamped mode. In each animal, only one successfully recorded cell with recognizable morphology was analyzed.

### Morphological Development of Biocytin-Filled Cells

After electrophysiological recordings, the electrode tip was gently withdrawn, and the slice was fixed with 4% (wt/vol) paraformaldehyde in 1XPBS at 4°C overnight. Thereafter, the slice was cut into 60-μm-thick sections with a vibratome as detailed previously (Zhan and Nadler, 2009). The yielded sections were orderly collected into 1XPBS. The sections were then incubated in 30% (vol/vol) methanol/2% (vol/vol) H_2_O_2_ for 90 min to inactivate endogenous peroxidases. After washing with 1X PBS, sections were incubated in an avidin-horseradish peroxidase solution at 4°C overnight. The avidin-horseradish peroxidase solution containing 0.3% (vol/vol) Triton X-100 was freshly prepared from the Vectastain Elite ABC Kit (Vector Laboratories, Burlingame, CA, USA). After washing again with 1XPBS, color was developed with diaminobenzidine/H_2_O_2_ for 6 min and intensified with nickel ammonium sulfate (DAB substrate kit, Vector Laboratories, Burlingame, CA, USA) according to the manufacturer’s instruction. Individual cells were classified according to their axonal projection domains as basket cells, HIPP (hilar cell with axonal terminals distributed in conjunction with perforant path termination field), HICAP (hilar cell with axon terminals in the commissural and association pathway termination field), and TML (hilar cell with axon terminals distributed in total molecular layer; Halasy and Somogyi, 1993; Han et al., 1993; Mott et al., 1997; Sík et al., 1997).

### Data Collection and Statistical Analysis

Quantitative data are expressed as mean ± standard error of the mean (SEM). Before subjecting to statistical comparisons, Kolmogorov-Smirnov test was conducted to confirm or to reject the normality of data sets. Data were statistically compared by Dunnett’s *post hoc* test following one-way analysis of variance (ANOVA), Bonferroni *post hoc* test following two-way ANOVA, unpaired *t*-test, or the Mann-Whitney test (the Wilcoxon rank sum test), as appropriate. *P* values less than or equal to 0.05 are considered to be statistically significant.

## Results

### Pilocarpine-Induced SE

Fourty rats were subjected to pilocarpine injection; all of them developed Racine stage II (*n* = 5) or higher (*n* = 35) seizures and eight rats died due to severe seizures. All deaths occurred within 30 min after the onset of seizures. SE in surviving pilocarpine-treated rats all lasted at least for 2 h. Twelve controls and 19 SE rats were used for determining IEG expression, whereas the rest of surviving rats were used for electrophysiological analysis. None of controls (*n* = 22) died until experiments were conducted.

### Arc and c-fos are Co-Expressed in the Same Cells with Parallel Intensity but Different Subcellular Localizations in Both the Resting and Stimulated Conditions

**Figure 1:**
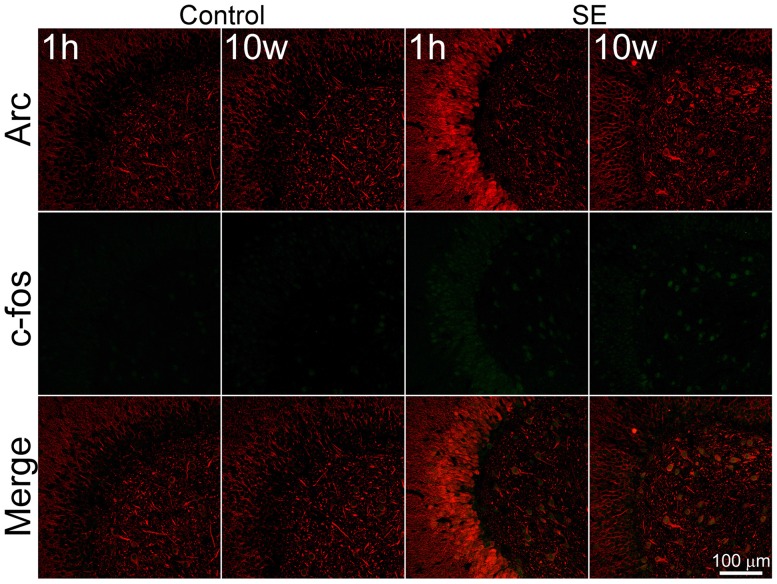
**Arc and c-fos are expressed in the same cells with parallel densities but different subcellular locations in both the controls and animals suffered from pilocarpine-induced status epilepticus (SE).** SE was induced by an intraperitoneal injection of pilocarpine and animals in the control group (Control) received equivalent amount of normal saline. Animals were sacrificed 1 h after the termination of a 2-h SE (indicated by 1 h) or more than 10 weeks after SE (indicated by 10 W). Horizontal hippocampal sections were double-stained with a goat anti-c-fos and rabbit anti-Arc and scanned with a confocal microscopy. Scale bar = 100 μm (applicable to all panels).

Despite the fact that both c-fos and Arc are widely used as molecular markers of neuronal activation (Kawashima et al., 2014; Yang et al., 2014), it remains to be known if expressions of these two markers are parallel at different stages after SE. We therefore co-stained Arc and c-fos to determine their expressions at different time points after pilocarpine-induced SE. In the controls sacrificed 1 h or more than 10 weeks after saline injection (Figure 1, left 2 columns), both c-fos and Arc were found to be expressed in the dentate gyrus at lower intensities in a cell type-dependent manner. Specifically, a portion of larger cells in the hilus was stained denser. In addition to their somatic expressions, both c-fos and Arc were expressed in the dendrites of granule cells, although the dendritic expression of Arc was far robust than that of c-fos. Within the soma, c-fos was expressed more intensely in the nucleus while Arc was more prominent in the cytoplasm with the exception of a few scattered cells in which the nucleic stain was very intense. Arc and c-fos were both up-regulated 1 h and more than 10 weeks after SE but the patterns of up-regulation at the two time points were quite different (Figure 1, right 2 columns). One hour after SE, cells with the highest intensity of Arc or c-fos were present in the granule cell layer (Figure 1, the 3rd column), whereas cells expressing Arc or c-fos with highest density more than 10 weeks after SE were present in the hilus (Figure 1, the 4th column).

### A Biphasic Increase in the Percentage of Hilar GABAergic Cells Positive for c-fos After Pilocarpine-Induced SE

**Figure 2:**
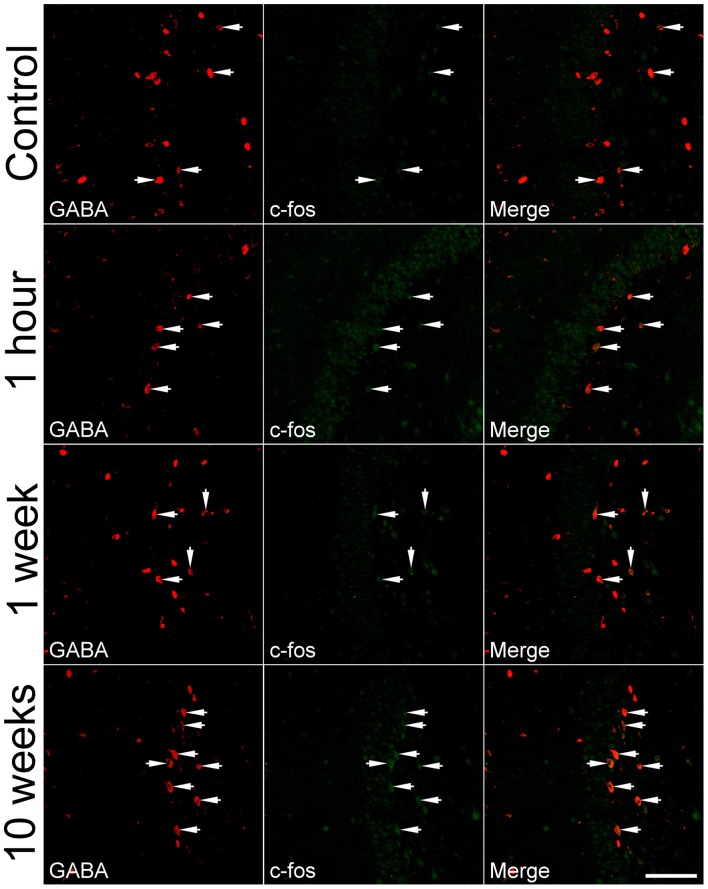
**Representative images show c-fos expression in GABA-immunoreactive cells in the dentate gyrus at 1 h, 1 week, and more than 10 weeks after SE.** SE was induced by an intraperitoneal injection of pilocarpine. The controls (Control) received equivalent volume of normal saline instead of pilocarpine. Left column: GABA immunoreactivity; Middle column: c-fos immunoreactivity; Right column: overlay of GABA and c-fos immunoreactivities. Arrows indicate GABA-positive cells with c-fos expression. Scale bar = 100 μm (applicable to all panels).

**Figure 3:**
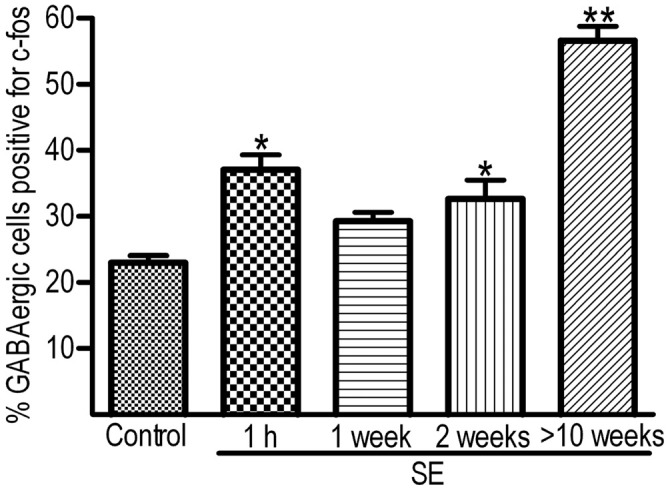
**Percentages of hilar GABAergic cells positive for c-fos at 1 h, 1 week, 2 weeks and more than 10 weeks after SE.** SE was induced by an intraperitoneal injection of pilocarpine. The controls (Control, *n* = 12) received equal volume of normal saline instead of pilocarpine. Because the percentages of GABAergic cells positive for c-fos in the controls examined at 1 h, 1 week, 2 weeks, or more than 10 weeks are not statistically different, data obtained at the above mentioned time points are pooled together. The number of animals in the SE group at 1 h, 1 week, 2 week and more than 10 weeks after SE onset is 4, 4, 4, and 7, respectively. Multiple comparisons were carried out with Dunnett’s test following one-way ANOVA. *p < 0.01 and **p < 0.001, as compared to “Control”.

To determine if IEGs are up-regulated in GABAergic cells after SE, c-fos was co-localized with GABA or GAD_67_. Representative images of the c-fos immunoreactivity in GABA-positive cells and quantitative data are shown in Figures 2, 3, respectively. In the controls at any time point, less than 30% of GABA-positive cells had c-fos expression (Figure 2, the upper row). Because no significant differences were revealed among those controls sacrificed at 1 h, 1 week, 2 weeks and more than 10 weeks, data obtained at above mentioned time points for the controls were pooled together and used for comparison to the SEs. One hour after SE, up-regulation of c-fos was found not only in granule cells but also in GABA-positive cells (Figure 2, 2nd row). The percentage of hilar GABAergic cells positive for c-fos increased from 23.0 ± 1.1% (*n* = 12) to 37.0 ± 2.3% (*n* = 4) [*p* < 0.01, Dunnett’s test following one-way ANOVA; *F*_(3,24)_ = 60.99, *p* < 0.0001]. Although there was a trend of c-fos up-regulation, data (*n* = 4) observed 1 week after SE (Figure 2, the 2nd row; Figure 3) were not significantly different from those of controls. Increased c-fos expression in GABAergic cells reemerged 2 weeks after SE (Figure 2, the 3rd row; Figure 3). More than 10 weeks after SE, c-fos expression in GABAergic interneurons became more frequent (Figure 2, the bottom row). Bar graphs comparing percentages of GABAergic cells positive for c-fos in the control and SE groups are shown in Figure 3. The number of animals in the SE group at 1 h, 1 week, 2 week and more than 10 weeks after SE onset is 4, 4, 4, and 7, respectively. Using GAD_67_ as a marker of GABAergic cells yielded similar results (Supplementary Figure 1).

### Up-Regulation of c-fos in GABAergic Interneurons is Subtype-Dependent

**Figure 4:**
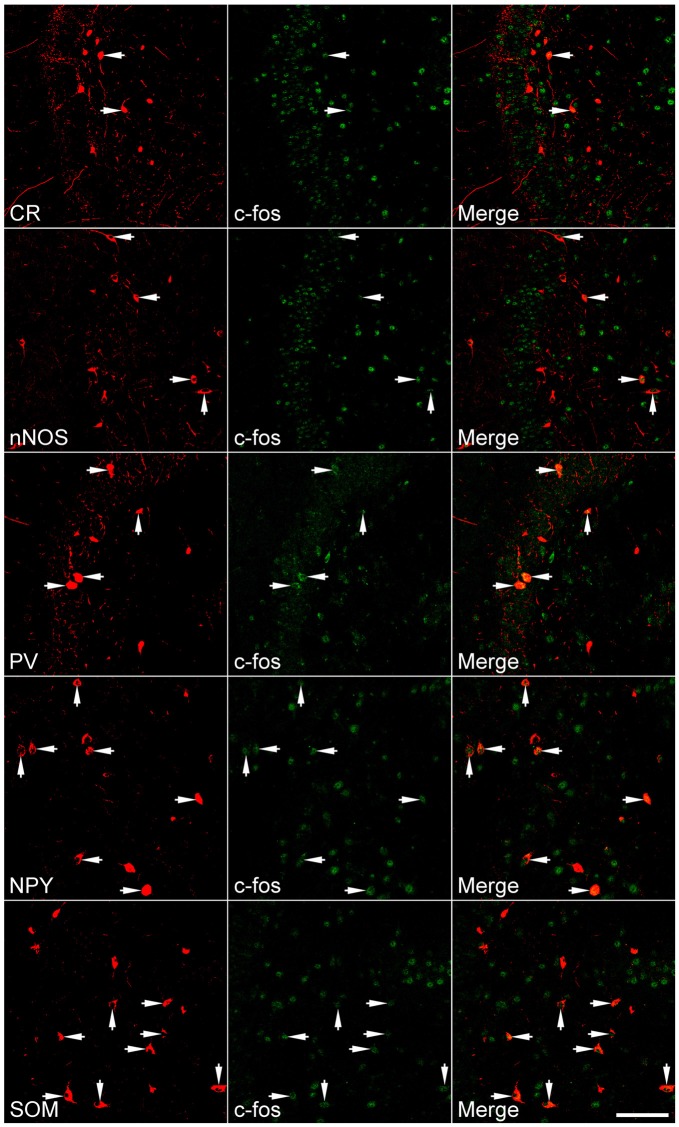
**Representative images show c-fos expression in neurochemically defined interneuron subtypes in the dentate gyrus more than 10 weeks after SE.** SE was induced by an intraperitoneal injection of pilocarpine. Left column: neurochemically defined interneuron subtypes (CR, calretinin; nNOS, neuronal nitric oxide synthase; NPY, neuropeptide Y; PV, parvalbumin; SOM, somatostatin); Middle column: c-fos immunoreactivity; Right column: overlay of marker and c-fos immunoreactivities. Arrows indicate marker-positive cells with c-fos expression. Scale bar = 100 μm (applicable to all panels).

**Figure 5:**
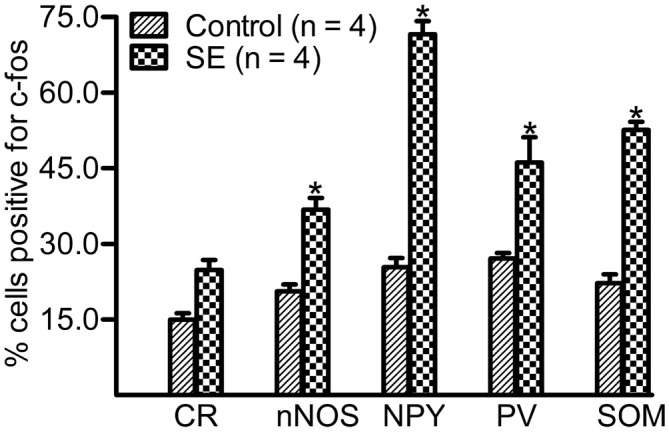
**Percentage of neurochemically defined hilar cells that express c-fos more than 10 weeks after SE.** SE was induced by an intraperitoneal injection of pilocarpine. The controls (Control) received equivalent volume of normal saline instead of pilocarpine solution. Sections prepared were double-stained with anti-c-fos and an anti-interneuron marker and were scanned with a Zeiss LSM780 confocal microscopy by using a 20× lens. Cells expressing c-fos in the marker-positive population were counted in three sections per rat by darkening hilar background stains. Data are statistically compared with Bonferroni *post hoc* test following 2-way ANOVA. Each data point is from four animals. *p < 0.01, compared to the Control.

Because a large portion of GABAergic cells expressed Arc and c-fos densely more than 10 weeks after SE, we attempted to determine if neurochemically defined GABAergic cells expressed IEGs in a subtype-dependent manner. To this end, c-fos was co-labeled with individual interneuron markers (CR, nNOS, NPY, PV, or SOM). The percentage of hilar GABAergic positive for c-fos among neurochemically defined subtypes more than 10 weeks after SE are shown in Figures 4, 5. In the controls, percentage of c-fos expression in neurochemically defined subtypes ranged from the highest 27.1 ± 1.1% in PV-positive cells to the lowest 14.9 ± 1.3% in CR-positive cells (Supplementary Figure 2 and Figure 5). More than 10 weeks after SE, with the exception of CR-positive cells, the percentage of GABAergic cells positive for c-fos significantly increased in all subtypes of GABAergic interneurons (Figures 4, 5). Specifically, the percentage differences between the controls and SEs in CR-, nNOS-, NPY-, PV-, and SOM-positive subtypes are 9.9, 16.3, 46.2, 19.0 and 30.3%, respectively.

### Most Hilar Interneurons Fire Spontaneously More than 10 Weeks After SE

**Figure 6:**
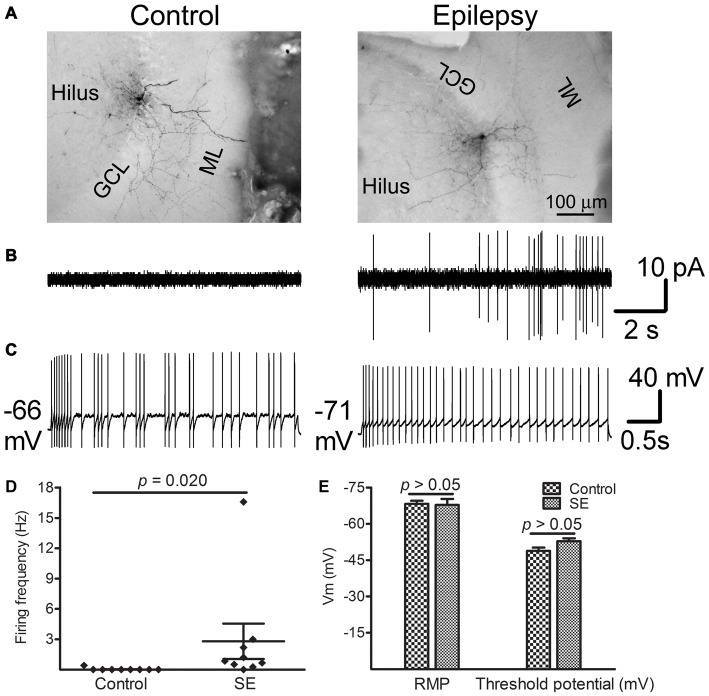
**Spontaneous and evoked firings recorded from morphologically identified hilar interneurons near the border of the granule cell layer and the hilus more than 10 weeks after SE.** Patch recordings were made in interneuron-like cells along the border between the granule cell layer and the hilus *in vitro*. After seals (>2 GΩ) were made, currents were recorded in cell-attached mode by holding cells at −60 mV for 5 min. Thereafter, the membrane was ruptured and whole-cell recordings were conducted. Resting membrane potential (REM) was read immediately after break-in. The threshold potential for firing was determined from stepped current injection. **(A)** Morphologically developed biocytin-filled cells in which electrophysiological recordings were made. Note that both cells are TML cells. **(B)** Traces show spontaneous firing obtained from morphologically identified cells shown in **(A)**. **(C)** Evoked firing in the same cells shown in **(A)** in response to current injection. **(D)** Difference in spontaneous firing frequency between control and SE groups. Diamonds represent the firing frequency in individual cells. The longer horizontal lines indicate the mean and the shorter horizontal lines indicate error bars in each group. Statistical analysis was conducted with the Mann-Whitney test. **(E)** Bar graphs compare RMP and the threshold potential between the control (*n* = 9) and SE (*n* = 9) groups. Statistical analyses were performed via unpaired *t*-test.

Figure 6 shows representative spontaneous and evoked firings in morphologically indentified hilar interneurons in the control and SE groups. Morphologies developed from biocytin-filling showed that the control group included one basket, three TML, three HICAP and two HIPP cells whereas the SE group had one basket, three TML, two HICAP and three HIPP cells in which only one HIPP cell did not fire spontaneously. Representative morphological images are shown in Figure 6A. Two out of nine interneurons recorded from the controls fired spontaneously in cell-attached mode at the near REM or even a 20 mV-depolarization from the REM. In contrast, eight out of the nine interneurons recorded more than 10 weeks after SE fired spontaneously (Figure 6B). Upon getting into the whole-cell mode, current injection evoked different firing patterns in individual cells (Figure 6C). Due to the limited number of cells recorded, a detailed analysis of evoked action potentials was not done. The spontaneous firing frequency at −60 mV was significantly higher in SE group (2.81 ± 1.75 Hz with a median of 0.84, *n* = 9) than that of the control group (0.05 ± 0.05 Hz with a median of zero, *n* = 9; *p* = 0.020 by the Mann-Whitney test; Figure 6D). The levels of REMs are −68.2 ± 1.3 mV in the control rats (*n* = 9) and −67.8 ± 2.5 mV in SE rats (*n* = 9; *p* > 0.05, by unpaired *t*-test), whereas the threshold potentials are −48.8 ± 1.3 mV in the controls (*n* = 9) and −52.9 ± 1.1 mV in the SE rats (*n* = 9; *p* > 0.05, by unpaired *t*-test; Figure 6E).

## Discussion

The major findings of the present study are that after a silent period following pilocarpine-induced SE, hilar GABAergic interneurons in the dentate gyrus persistently express IEGs in a subtype-dependent fashion, and in addition, most GABAergic cells along the border between the granule cell layer and the hilus fire spontaneously at near REM.

### c-fos and Arc Can be Used Interchangeably as Molecular Markers of Neuronal Activity in GABAergic Interneurons After Pilocarpine-Induced SE

At least four IEGs including c-fos, Arc, Egr1 (Zif268) and Npas4 are known to be up-regulated transiently by seizures (Simonato et al., 1991; Lerea et al., 1992; Link et al., 1995; Lyford et al., 1995; Honkaniemi and Sharp, 1999; Akiyama et al., 2008; Dyrvig et al., 2012; Szyndler et al., 2013; Wang D. et al., 2014). It is unclear if expression of those IEGs can be used to indicate activity of GABAergic cells over the course of SE progressing to spontaneous seizures, due to at least three reasons. First, evidence indicates that activity-dependent genes are differentially regulated in different cell types by the same stimulus (Lyons and West, 2011). Second, expression of certain IEGs may vary between GABAergic cells and excitatory neurons (Vazdarjanova et al., 2006). In addition, IEGs may be differentially up-regulated in response to different stimuli in the same cell type (Ramamoorthi et al., 2011; Spiegel et al., 2014). We did two sets of co-localization at different time points after pilocarpine-induced SE to eliminate those concerns. The c-fos/Arc co-localization experiment clearly revealed that c-fos and Arc had a parallel expression at all time points examined in all neuron types in the dentate gyrus, implying that both c-fos and Arc could be used interchangeably as indicators of neuronal activation after pilocarpine-induced SE. This finding is in agreement with the conclusion drawn from a study done with double-fluorescence *in situ* hybridization (Pinaud et al., 2008). Furthermore, the c-fos/GABA co-localization experiment demonstrated that up-regulation of c-fos in hilar GABAergic interneurons after SE is biphasic, where an earlier phase up-regulation was followed by a short silent period, and then a persistent up-regulation emerged.

### After a Silent Period Following Pilocarpine-Induced SE, Hilar GABAergic Interneurons Become Persistently Hyperactive

Behavioral and electrographic changes in the pilocarpine rat model of epilepsy consist of three distinct periods: an initial period that builds up rapidly into SE is followed by a silent period varying from 1 to 6 weeks and later by the appearance of spontaneous recurrent seizures (Cavalheiro, 1995; Curia et al., 2008). In contrast to other chronic epilepsy models, spontaneous seizures recur frequently and consistently in virtually all pilocarpine-treated rats (Biagini et al., 2013). Neuronal damage after systemic administration of pilocarpine exists in multiple brain structures such as the olfactory cortex, amygdala, thalamus, neocortex, hippocampal formation, and substantia nigra (Fujikawa, 1996; Curia et al., 2008; Furtado et al., 2011). Within the hippocampal formation, neuronal damage after pilocarpine-induced SE is region-, time-, and cell type-dependent. One report showed the number of hilar neurons down by approximately 50% which consisted of both mossy cells and interneurons (Cardoso et al., 2011). The loss of GABAergic interneurons contains different neurochemically defined subtypes (André et al., 2001) and peaks within days after SE onset (André et al., 2001; Castro et al., 2011).

An hour after termination of SE, nearly 40% of GABAergic interneurons became c-fos/Arc-positive. One week after SE, the percentage of hilar GABAergic interneurons positive for c-fos was not significantly higher than that of controls; however, the up-regulation of c-fos resurfaced 2 weeks after SE. More than 10 weeks later, nearly 60% of GABAergic cells expressed c-fos, and surprisingly the intensity of c-fos expression in many GABAergic interneurons exceeded those neighborhood granule cells. The nearly normal expression of IEGs found 1 week after SE is not surprising, since a latent period after pilocarpine-induced SE in the rat has been reported to be between 7–18 days (Cavalheiro et al., 1987, 1991; Priel et al., 1996; Goffin et al., 2007). To support this further, an electrophysiological study showed a reduced excitatory drive onto dentate GABAergic interneurons from day one through day 8 after pilocarpine-induced SE (Doherty and Dingledine, 2001). Thus, the overall expression pattern of IEGs after pilocarpine-induced SE correlates well to the progression of epilepsy.

It has been well demonstrated that a rise of calcium in the nucleus is critical in up-regulation of IEGs after neuronal activity. This increase in intracellular calcium can be achieved through multiple routes, such as opening of voltage-gated calcium channels in response to membrane depolarization, glutamate receptor activation, or calcium release from intracellular stores (Hagenston and Bading, 2011; West and Greenberg, 2011). Direct activation of muscarinic acetylcholine receptors on interneurons (Soulé et al., 2012; Yi et al., 2015) and enhanced excitatory neurotransmission (Simonato et al., 1991; Lerea et al., 1992) might contribute to the earlier phase of IEG up-regulation. The late phase of IEG up-regulation after SE has rarely been reported, even in excitatory neurons. It is unlikely that the up-regulation of IEGs occurred after spontaneous seizure episodes. First, it was observed in every animal more than 10 weeks SE. Second, different from what happened at 1 h after SE, the intensity of c-fos immunoreactivity in most GABAergic cells exceeded that in neighborhood granule cells. Third, instead of PV-positive cells which are known to provide acute and quick inhibition against increased excitatory synaptic transmission, NPY- and SOM-positive cells displayed most extensive expressions. Forth, the increased spontaneous firing was observed in nearly 90% of interneurons *in vitro* several hours after they have been obtained. Last, recent *in vivo* electrophysiological and/or optogenetic studies clearly showed that increased interneuronal firing preceded the occurrence of spontaneous seizures (Gnatkovsky et al., 2008; Ellender et al., 2014; Uva et al., 2015; Yekhlef et al., 2015). Mechanisms underlying the late phase up-regulation of IEGs after SE may be multiple, specifically calcium-dependent and/or neurotrophic factors-driven. However, given the evidence that brain-derived neurotrophic factor (BDNF) was not up-regulated in GABAergic interneurons after neuronal stimulation (Spiegel et al., 2014), it is unlikely that neurotrophic factor pathway was primarily involved. Electrophysiological recordings showed that nearly 90% of morphologically proved hilar GABAergic interneurons displayed intense spontaneous firing. Despite spontaneous firing was not recorded, a newly published study conducted in the same epilepsy model showed that the intrinsic membrane properties and evoked firings were not different between the control and SE in HICAP and TML cells (Yu et al., 2016). On the basis that neither the REM nor the threshold potential for action potential differed from controlled animals, the increased spontaneous firing was probably not due to altered intrinsic membrane properties but some other mechanism(s). A plausible candidate would be the increased synaptic excitation-inhibition ratio, as it has been demonstrated in hippocampal CA1 region regardless of specific types of interneurons (Stief et al., 2007) and in hilar SOM-positive cells (Zhang et al., 2009; Halabisky et al., 2010). The increased synaptic excitation-inhibition ratio could result in glutamate receptor activation and subsequent calcium influx, contributing to up-regulation of IEGs. It is noticeable that due to few cells were recorded in both groups and the expression of IEGs in individual cells was unmeasured, how the expression of IEGs correlated to spontaneous firing in a single cell could not be established. Future studies combing electrophysiology and single cell RNA-sequencing would be useful to solve this issue (Muñoz-Manchado et al., 2015; Fuzik et al., 2016).

### Persistent Hyperactivity of Hilar GABAergic Interneurons After the Silent Period Following SE is Subtype-Dependent

Several subtypes of neurochemically defined GABAergic interneurons exist in the dentate gyrus; they are not only different in morphological appearances, electrophysiological properties, and axonal projection destinations, but also in distribution pattern and density. Regardless of overlapping, cells expressing SOM, NPY, nNOS, CR and PV are the most commonly seen subtypes in the rodents (Jinno and Kosaka, 2006; Houser, 2007; Liang et al., 2013). Different subtypes originate in different parts of the developing ventral telencephalon and may be complementary in network computation and control of activity states (Kvitsiani et al., 2013).

It is widely accepted that different neurochemically defined subtypes of GABAergic interneurons respond to SE differently. For example, SOM- and PV-positive cells decrease in number in response to SE, although PV-positive cells are lost to a lesser extent (Houser, 2014). Behaviors of the other subtypes are less defined. In addition to the loss of GABAergic interneurons, structural and functional plasticity also occurs in surviving GABAergic cells in this area. After pilocarpine-induced SE in the rat, somata of surviving SOM-positive cells appear to be enlarged, dendrites become lengthened morphologically, and the excitatory drive onto SOM-positive cells is increased (Halabisky et al., 2010).

There have been studies showing that c-fos expression is increased shortly after a transient spontaneous seizure in PV-expressing cells (Scharfman et al., 2002; Peng and Houser, 2005). Activity of other subtypes of GABAergic interneurons has rarely been investigated. In the present study, we found that c-fos was strongly up-regulated in SOM- and NPY-positive cells, moderately increased in PV- and nNOS-positive neurons, and lightly changed in CR-positive cells. The up-regulation seen in SOM-positive cells corresponds to their heavy losses and robust plasticity in response to SE (Zhang et al., 2009; Halabisky et al., 2010). In relation to epilepsy, NPY-positive cells in the dentate gyrus have not been studied as extensively as SOM-positive cells. It is thus surprising that the percentage of NPY-positive cells expressing c-fos was far higher than other neurochemically defined subtypes of interneurons. A study done in humans with temporal lobe epilepsy found similar degrees of loss of SOM- and NPY-positive dentate interneurons (Sundstrom et al., 2001). In the kainate-induced SE model, NPY-positive cells were found to be relatively resistant to SE compared to PV-positive cells (Kuruba et al., 2011). To our knowledge, there have been no electrophysiological studies that explored the functional changes in NPY-positive interneurons in the dentate gyrus after SE. However, NPY-positive cells were found to undergo intense structural plasticity in a time course similar to SOM-positive cells (Drexel et al., 2012). Given that both SOM- and NPY-positive cells become hyperactive and hyperplastic, it is reasonable to speculate that the robust plasticity and hyperactivity are closely related. In comparison to NPY- or SOM-positive cells, percentage of PV-positive cells expressing c-fos was lower. As potent calcium buffering exists in PV-positive cells (Baimbridge et al., 1992), it remains arguably that the lower c-fos expression in this interneuron subtype may be caused by potent calcium buffering. Because few studies have dealt how CR-positive cells would respond to SE, it is currently impossible to say why few CR-positive cells expressed c-fos in animals with spontaneous seizure. Since nNOS is most frequently co-localized with CR (Liang et al., 2013), it is not supersizing that the expression of IEGs is also lower in nNOS-positive cells.

### Significances and Implications of Persistent Hyperactivity of Interneurons After SE

Under normal conditions, the dentate gyrus acts as a gate in filtering excitatory information from upstream of the entorhinal cortex to downstream of the CA3 region. A persistent hyperactivity of GABAergic cells after SE may reflect a fragile balance between excitation and inhibition in the dentate network. As a result, a sudden increase in excitation or a decrease in inhibition could allow seizures to occur. It also implies that strengthening inhibition is needed in preventing the occurrence of spontaneous seizures. Because transplantation of GABAergic precursors has been shown to be remarblely effective in reducing the frequency of spontaneous seizures in epileptic animals (Calcagnotto et al., 2010; Hunt et al., 2013; Henderson et al., 2014; Southwell et al., 2014) and because the persistent hyperactivity of GABAergic cells is subtype-dependent, it should be tested if transplantation with one subtype of GABAergic precursors would be more effective than another. On the other hand, the same mechanism(s) underlying the persistent hyperactivity of GABAergic interneurons may drive the structural and functional plasticity to occur. Finally, a persistent hyperactivity of GABAergic interneurons could serve as a mechanism in contributing to epileptiform activity, because a persistent activation of GABA receptors in principle cells would lead intracellular chloride to rise, resulting in positive shifts in the GABAergic reversal potential (Fujiwara-Tsukamoto et al., 2007). Positive shifts in the GABAergic reversal potential in a heterogeneous neuronal population due to intracellular chloride accumulation in combination with other pathological events could result in ictogenesis (Alfonsa et al., 2015).

## Author Contributions

XW: designed and performed research, wrote the article. XS: animal modeling. LW: analyzed data. JVN: designed research. R-ZZ: designed research and wrote the article.

## Conflict of Interest Statement

The authors declare that the research was conducted in the absence of any commercial or financial relationships that could be construed as a potential conflict of interest. The reviewer ER and handling Editor declared their shared affiliation, and the handling Editor states that the process nevertheless met the standards of a fair and objective review.
