# Supplementary material for: Persistent Hyperactivity of Hippocampal Dentate Interneurons After a Silent Period in the Rat Pilocarpine Model of Epilepsy
Source: Front Cell Neurosci. 2016 Apr 8;10:94. doi: 10.3389/fncel.2016.00094 (PMC4824773; doi:10.3389/fncel.2016.00094)
Supplement: Supplementary file 1 [file DataSheet_1.pdf]

Supplementary Material

Persistent Hyperactivity of Hippocampal Dentate Interneurons after a Silent Period in the Rat Pilocarpine Model of Epilepsy

Xiaochen Wang[1], Xinyu Song[2], Lin Wu[1], J Victor Nadler[3], Ren-Zhi Zhan[1*]

[1] Department of Physiology, Shandong University School of Medicine, Jinan, China

[2] Department of Respiratory Medicine, Affiliated Hospital of Binzhou Medical University, Binzhou, Shandong, China

[3] Department of Pharmacology and Cancer Biology, Duke University Medical Center, Durham, NC, USA

*** Correspondence:** Dr. Ren-Zhi Zhan, Department of Physiology, Shandong University School of Medicine, Jinan 250012, China

zhan0001@sdu.edu.cn

1. Supplemental Fig. 1.

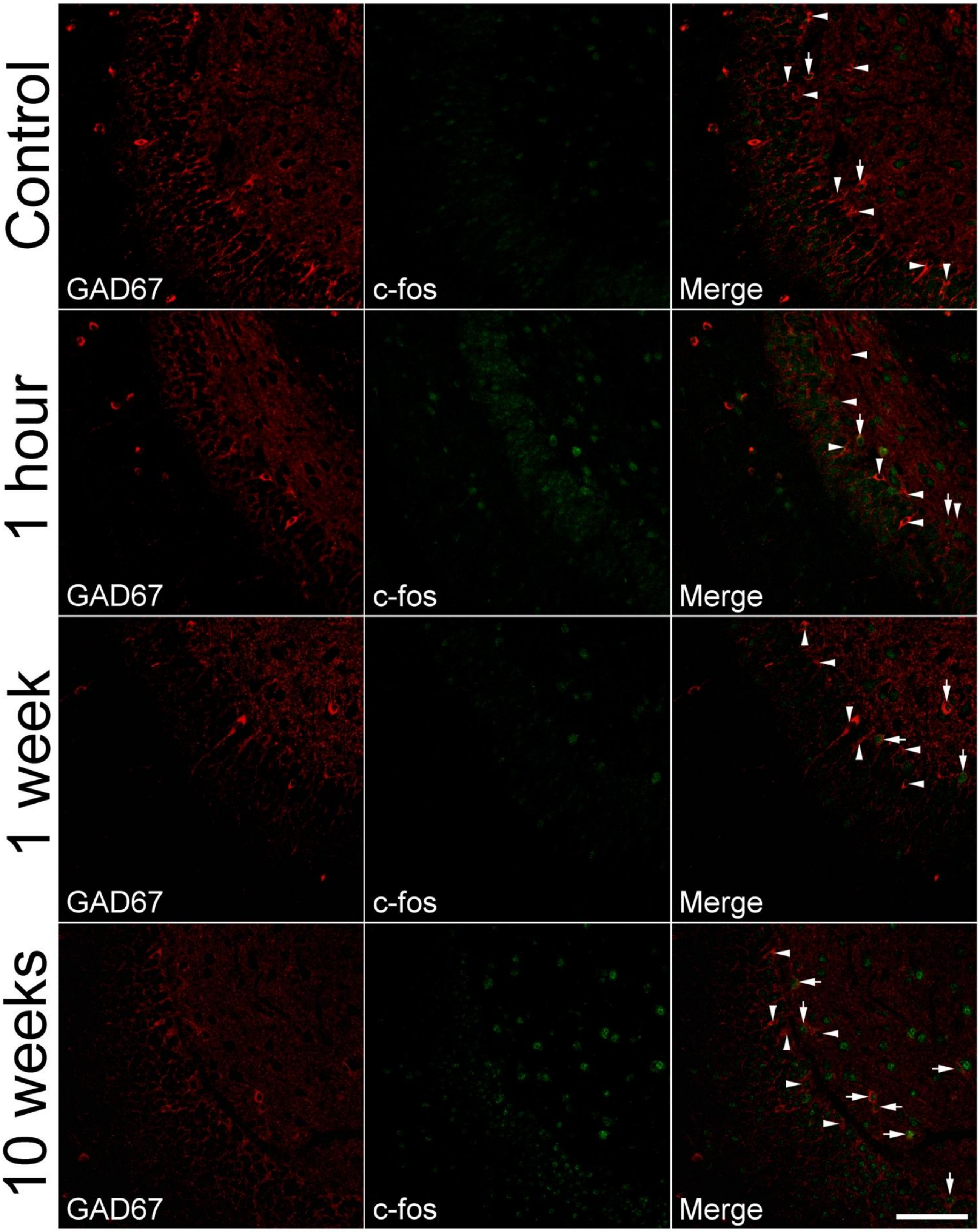
Control
1 hour
1 week
10 weeks
GAD67
c-fos
Merge
GAD67
c-fos
Merge
GAD67
c-fos
Merge
GAD67
c-fos
Merge

Fig.1. Representative images show c-fos expression in GAD_{67}-immunoreactive cells in the dentate gyrus 1 hour, 1 week, two weeks and more than 10 weeks after SE

SE was induced by an intraperitoneal injection of pilocarpine. The controls (Control) received equivalent volume of normal saline instead of pilocarpine solution. Hilar cells positive for GAD_{67} are indicated by arrows. Arrowheads indicate GAD_{67}-positive cells without c-fos expression. Left column: GAD_{67} immunoreactivity; Middle column: c-fos immunoreactivity; Right column: overlay of the left and middle columns. Scale bar = 100 μm (applicable to all panels).

2. Supplemental Fig. 2.

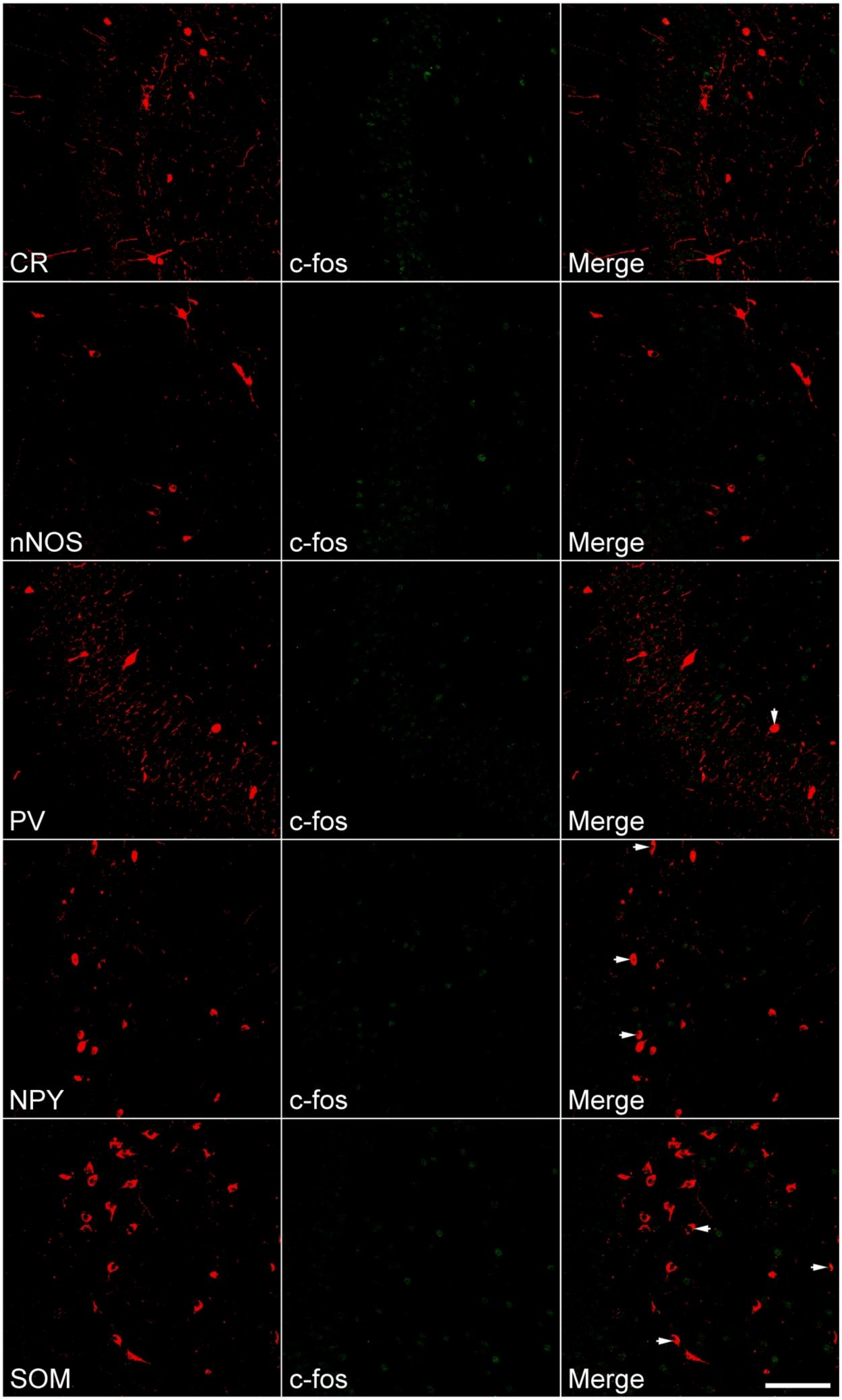
CR
c-fos
Merge
nNOS
c-fos
Merge
PV
c-fos
Merge
NPY
c-fos
Merge
SOM
c-fos
Merge

Fig. 2. Representative images show c-fos expression in neurochemically defined interneuron subtypes in the dentate gyrus of the controls (Control)

Instead of pilocarpine, the controls (Control) received equivalent volume of saline. Left column: neurochemically defined interneuron subtypes (CR: calretinin; nNOS: neuronal nitric oxide synthase; NPY: neuropeptide Y; PV: parvalbumin; SOM: somatostatin); Middle column: c-fos immunoreactivity; Right column: overlay of the left and middle columns. Arrows indicate marker-positive cells with c-fos expression. Scale bar = 100 μm (applicable to all panels).
